# Effect of vitamin B1 supplementation on bone turnover markers in adults: an exploratory single-arm pilot study

**DOI:** 10.1017/jns.2025.22

**Published:** 2025-05-08

**Authors:** Akinori Hara, Chie Takazawa, Hiromasa Tsujiguchi, Jiaye Zhao, Masaharu Nakamura, Tomoko Kasahara, Yukari Shimizu, Hiroyuki Nakamura

**Affiliations:** 1 Department of Hygiene and Public Health, Faculty of Medicine, Institute of Medical, Pharmaceutical and Health Sciences, Kanazawa University, Kanazawa, Ishikawa, Japan; 2 Department of Nursing, Faculty of Health Sciences, Komatsu University, Komatsu, Ishikawa, Japan

**Keywords:** Nutrition, Osteoclast, Osteoporosis, Prevention, Thiamine, ,BMD, bone mineral density, ,BMI, body mass index, ,eGFR, estimated glomerular filtration rate, ,P1NP, N-terminal propeptide of type I procollagen, ,PTH, parathyroid hormone, ,TRACP, tartrate-resistant acid phosphatase, ,25(OH)D, 25-hydroxyvitamin D

## Abstract

Although B vitamins have been shown to play beneficial roles in bone health, the effects of vitamin B1 in humans are still unclear. This study aimed to investigate the effects of vitamin B1 supplementation on middle-aged and older adults. This single-armed trial study included community-dwelling adults in Japan and used a pre- and post-test design. The participants were given 28.0 mg of vitamin B1 supplementation per day for 1 month in addition to their daily usual diet. The effect of this treatment on bone turnover markers and metabolism was evaluated at baseline and after 1 month. Forty-two participants were enrolled (mean age, 58.6 ± 10.4 years; 36 women). The vitamin B1 levels in whole blood increased significantly from baseline after vitamin B1 supplementation. The level of serum tartrate-resistant acid phosphatase 5b (TRACP 5b), a bone resorption marker, reduced significantly (378 ± 135 vs. 335 ± 120 mU/dL, p < 0.001), while the level of N-terminal propeptide of type I procollagen (P1NP), a marker specific to bone formation, did not change. Moreover, the serum phosphorus and parathyroid hormone (PTH) concentrations did not change, whereas the corrected serum calcium concentrations increased and vitamin D concentrations decreased. The serum TRACP 5b levels decreased after vitamin B1 supplementation in the middle-aged and older adults. Further definitive trials are needed to determine the efficacy of vitamin B1 in improving bone health.

Fractures associated with falls and/or osteoporosis, particularly in the elderly, are an important public health issue. Globally, while an estimated 684,000 fatal falls occur each year, approximately 37.3 million falls severe enough to require medical attention occur each year, increasing the years lived with disability.^(1)^ Among those at risk for falls, the elderly tend to have a higher risk, especially women over 55 years of age and men over 65 years of age, with a progressive increase in fractures complicated by osteoporosis.^(2)^ In Japan, about 12.8 million people have osteoporosis, which corresponds to approximately 10% of the total population, and approximately 193,400 fractures occur annually.^(3)^ Of note, another study showed that 79.5% of hip fractures were caused by falls from a standing height or from a bed.^(4)^

Among prevention strategies at individual levels of both falls for older people and osteoporosis, especially for the latter, good nutrition in addition to regular physical activities and avoidance of harmful lifestyle habits are recommended for all people at risk.^(2,5)^ From a nutritional perspective, a balanced energy and nutrient intake have been recommended as a basic preventive and therapeutic measure against osteoporosis. Current guidelines recommend adequate daily calcium and vitamin D intake in postmenopausal women, but the efficacy of supplementation in fracture reduction needs to be investigated further.^(2,5)^ In addition to these established nutritional elements, increasing evidence has shown that an adequate intake of B vitamins involved in homocysteine metabolism, such as vitamin B6, vitamin B12, and folic acid, is also necessary to maintain good bone health.^(6–8)^

Of all the B vitamins, vitamin B1 is considered an essential cofactor for key enzymes involved in energy metabolism and neurotransmitter synthesis.^(7,8)^ To date, only limited evidence can confirm the relationship between vitamin B1 and bone health. One observational study has shown that thiamine deficiency was observed throughout the postoperative period in patients with femoral neck fracture.^(9)^ Another observational study has shown that dietary thiamine intake was not associated with hip fracture risk in women and men.^(10)^ Recently, in vitro and in vivo experiments have reported that thiamine diphosphate (ThDP), a major vitamin B1 derivative, may be involved in bone homeostasis, especially in regulating osteoclast differentiation and function.^(11)^ Mechanistically, ThDP is considered to be protective in cells by regulating reactive oxygen species and unfolded protein response,^(12)^ suggesting its potential role in protecting bone loss as well as other cell types such as neurons. However, the importance of vitamin B1 in the bone health of humans needs to be investigated further.

The aim of this pilot study is to assess the effects of vitamin B1 supplementation on the biochemical markers of bone turnover and metabolism in community-dwelling middle-aged and older adults.

## Methods

### Study design and participants

This non-randomised, single-arm pilot trial recruited participants who had lived in Shika town, Ishikawa, Japan, between December 1 and 31, 2022. The inclusion criteria were as follows: age ≥ 40 years and an estimated glomerular filtration rate (eGFR; calculated using the equation in the ‘Procedures’ section) of ≥ 60 mL/min/1.73 m^2^. On the other hand, the exclusion criteria were as follows: receiving regular anti-osteoporosis medications; supplementation of calcium, vitamin D, or vitamin K; current or past glucocorticoid treatment for underlying diseases; severe liver disease; history of fractures within 6 months prior to enrollment; history of gastrointestinal surgery; or receiving treatment for malignancies. Because of the exploratory nature of the study, individuals of any sex and age who were interested in bone health were eligible to participate as long as they met these inclusion criteria and did not violate the exclusion criteria. This trial was conducted in accordance with the Declaration of Helsinki and was approved by the Medical Ethics Committee of Kanazawa University (No. 114066-1). Furthermore, all patients provided written informed consent before starting the trial.

### Procedures

After enrollment, all participants received oral vitamin B1 (thiamine) supplement (Nature Made B-1, Otsuka Pharmaceutical Co., Ltd., Tokyo, Japan) at a daily recommended dose of 28.0 mg (2 tablets) for 1 month, which was added to their usual daily diet. This tablet form, which is commercially available as a daily dose within the range approved by authorities for the prevention and treatment of vitamin B1 deficiency,^(13)^ was considered convenient and safe for the study participants. Furthermore, referring to a previous study of vitamin B12 and folate,^(14)^ in which the effects of these vitamins on bone turnover markers were investigated, one month, the timeframe at which the increase in blood levels of the marker had reached a plateau, was set as the intervention period in the present study. Adherence to the supplements was assessed by asking the participants to return the containers used at the end of the study along with their self-reports. As an additional reference finding to confirm that the supplement was taken, total thiamine levels in whole blood were measured using liquid chromatography-tandem mass spectrometry (SRL, Inc., Tokyo, Japan) at baseline and at 1 month (Supplementary information).^(15)^ The assessments of biochemical response were performed at baseline and 1 month after supplementation. During this period, participants were instructed to continue their usual diet and not to add any medications or supplements.

The anthropometric parameters were measured at baseline, and non-fasting blood specimens were collected between 1400 and 1700 at baseline and at 1 month of follow-up. The serum creatinine levels were measured using the enzymatic method and used to calculate the eGFR using the following equation for Japanese patients: eGFR (mL/min/1.73 m^2^) = 194 × serum creatinine^−1.094^ × age^−0.287^ (if female, × 0.739).^(16)^ The level of serum TRACP 5b as a bone resorption marker was measured using enzyme immunoassay (N-test, Nittobo Medical Co., Ltd., Fukushima, Japan), while the level of P1NP, a marker specific to bone formation, was measured using electrochemiluminescence immunoassay (Cobas, Roche Diagnostics K.K., Tokyo, Japan) by SRL, Inc. (Tokyo, Japan). Moreover, serum was tested for calcium, phosphate, albumin, PTH (measured using electro chemiluminescence immunoassay) (Cobas, Roche Diagnostics K.K.), and 25-hydroxyvitamin D [25(OH)D] (measured using chemiluminescent enzyme immunoassay) (Lumipulse, Fujirebio Inc., Tokyo, Japan) by SRL, Inc. Moreover, the serum calcium concentrations were corrected for albumin.

### Other variables

The body composition parameters at baseline were measured using a body composition analyser (MC-780A-N, Tanita Co. Ltd., Tokyo, Japan), and the body mass index (BMI) was calculated as weight (kg) divided by [height (m)]^2^. Other variables, such as age, sex, smoking status, frequency of exercise, and alcohol consumption, were assessed using self-administered questionnaires. Smoking status was classified as current or non-current smoker (non-smoker or past smoker), and habitual alcohol consumption was defined as drinking more than one glass of Japanese sake (22-g ethanol) per day at least three times a week.^(17)^ The frequency of exercise was estimated as follows: the participants were asked whether they had exercised for more than 30 min at least twice a week or had performed tasks, such as walking, cleaning, and carrying baggage for more than 1 h per day.^(17)^ The participants who responded affirmatively to any of these questions were considered to have a habit of performing physical activities at an adequate level based on the World Health Organization guidelines on physical activity.^(18)^

### Outcomes

The primary efficacy endpoint was the change in the TRACP 5b and P1NP levels from baseline at 1 month after supplementation. The secondary endpoints included changes from baseline in the serum calcium, phosphate, PTH, and 25(OH)D levels from baseline at 1 month.

### Statistical analysis

In this pilot study, the sample size calculation was performed using the G power 3.1 software.^(19)^ When testing the difference in the mean serum bone turnover marker concentrations before and after vitamin B1 supplementation, the total sample size was 34 cases with an effect size of 0.25, significance level of 5%, and power of 0.80. Based on the calculation, the target number of cases was set at 40, considering a dropout rate of approximately 20%. Moreover, all participants were included in the statistical analysis. The continuous variables were expressed as means with standard deviations, whereas the categorical variables were expressed as numbers with proportions. After confirming that TRACP 5b was normally distributed, the differences in the pre- and post-intervention whole blood levels of vitamin B1, biomarkers of bone turnover, and metabolism for the entire population were evaluated using paired *t*-tests. The subgroup analysis according to the baseline characteristics was conducted using a mixed-effects model to estimate the change from baseline to 1 month after the measurement of the outcome variables. Age and sex were included as covariates in the model, while intergroup differences were compared with a two-sided significance level of 0.05. All statistical analyses were performed using SPSS version 28 (IBM Corp., Tokyo, Japan).

## Results

### Characteristics of the study participants

The study participants began receiving the required supplements in January 2023, which was completed in February 2023. In total, 42 participants were enrolled and completed the study (Supplementary Fig. 1). As shown in Table 1, the mean age of the participants was 58.6 ± 10.4 years, and 36 of the total study population were women. The mean BMI was 21.5 ± 2.7 kg/m^2^, and the mean eGFR was 80.0 ± 12.1 ml/min/1.73m^2^.

**Table 1.** Characteristics of the study participants (n = 42)

	Overall (n = 42)		Men (n = 6)		Women (n = 36)	
	Mean/n	SD/%	Mean/n	SD/%	Mean/n	SD/%
Age, years	58.6	10.4	63.5	8.1	57.8	10.6
History of fracture, n	10	23.8	2	33.3	8	22.2
Drinking habit, n	17	40.5	5	83.3	12	33.3
Current smoking, n	1	2.4	1	16.7	0	0
Physical activity, n	21	50	3	50.0	18	50.0
Diabetes, n	1	2.4	1	16.7	0	0
Hypertension, n	3	7.1	1	16.7	2	5.6
Dyslipidemia, n	4	9.5	0	0	4	11.1
BMI, kg/m^2^	21.5	2.7	24.3	1.9	21.1	2.5
HbA1c, %	5.4	0.3	5.4	0.5	5.4	0.3
eGFR, ml/min/1.73 m^2^	80.0	12.1	75.6	6.7	80.8	12.7
Serum-corrected calcium, mg/dL	9.3	0.3	9.2	0.3	9.3	0.3
Serum phosphate, mg/dL	3.5	0.5	3.3	0.6	3.5	0.5

Data are expressed as means, SD or n, %.

SD, standard deviation; BMI, body mass index; HbA1c, glycated haemoglobin; eGFR, estimated glomerular filtration rate.

With regard to treatment adherence, all 42 participants reported that they ingested the required doses of the supplements and returned the containers used at the end of the study. Moreover, the mean plasma concentrations of vitamin B1 at baseline and 1 month after starting the supplementation were 32.2 ± 6.1 ng/mL and 44.7 ± 8.1 ng/mL (p < 0.001), respectively (Table 2).

**Table 2.** Changes in the biomarkers of bone turnover and metabolism

	Pre		Post		p value
	Mean	SD	Mean	SD	
Vitamin B_1_, ng/mL	32.2	6.1	44.7	8.1	**< 0.001**
TRACP 5b, mU/dL	378.0	134.5	335.4	120.1	**< 0.001**
P1NP, ng/mL	51.5	18.2	53.0	16.3	0.244
Serum-corrected calcium, mg/dL	9.3	0.3	9.5	0.3	**< 0.001**
Serum phosphate, mg/dL	3.5	0.5	3.5	0.6	0.634
Serum PTH, pg/mL	46.3	16.6	45.0	14.3	0.483
Serum 25(OH)D, ng/mL	16.0	5.7	15.2	5.9	**0.015**

Data are expressed as mean, standard deviation (SD). The comparisons between pre- and post-supplementation were performed using the paired *t-test*. p-values < 0.05 are highlighted in bold.

TRACP 5b, tartrate-resistant acid phosphatase 5b; P1NP, N-terminal propeptide of type I procollagen; PTH, parathyroid hormone; 25(OH)D, 25-hydroxyvitamin D.

### Changes in the bone turnover markers

**Fig. 1.**
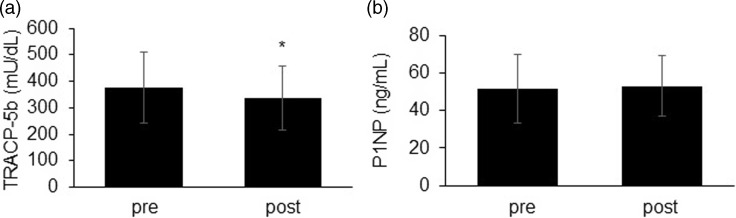
Change in the levels of bone turnover markers. (a) TRACP 5b. (b) P1NP. *p < 0.001. TRACP 5b, tartrate-resistant acid phosphatase 5b; P1NP, N-terminal propeptide of type I procollagen.

Table 2 and Fig. 1 show the changes in the serum TRACP 5b and P1NP levels from baseline at 1 month after supplementation. The mean baseline levels of TRACP 5b were 378.0 ± 134.5 mU/dL, and these values significantly reduced to 335.4 ± 120.1 mU/dL (Fig. 1a). Furthermore, no significant change in the serum P1NP concentration was observed (Fig. 1b).

### Changes in other biomarkers associated with bone metabolism

As shown in Table 2, the serum phosphate and PTH levels were not changed after vitamin B1 supplementation compared with the baseline values, whereas the corrected serum calcium concentration significantly increased at 1 month (9.3 ± 0.3 vs. 9.5 ± 0.3 mg/dL). Moreover, the serum 25(OH)D levels significantly decreased from baseline at 1 month (16.0 ± 5.7 vs. 15.2 ± 5.9 ng/mL).

### Subgroup analysis

**Fig. 2.**
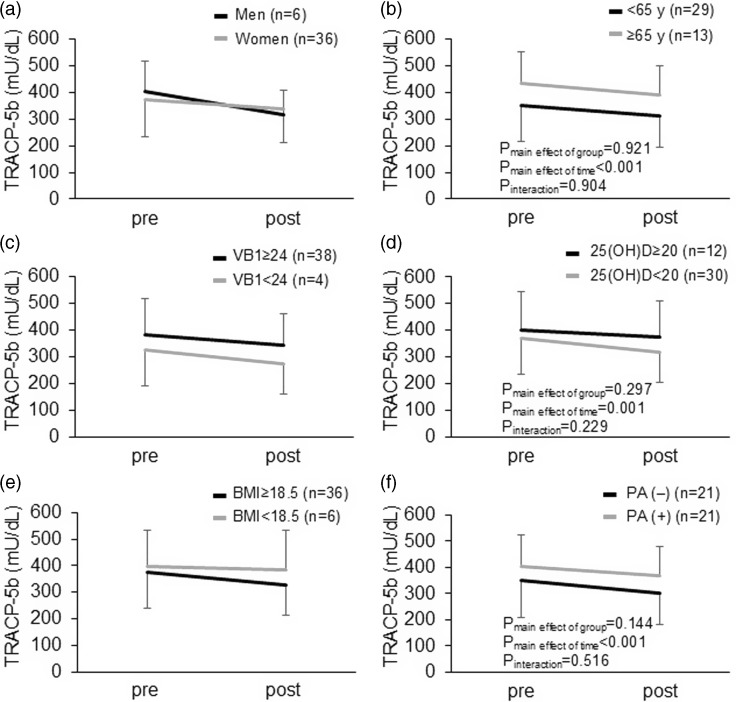
Subgroup analysis for the changes in the serum TRACP 5b levels. (a) Sex. (b) Age. (c) Baseline VB1 levels in whole blood. (d) Baseline serum 25(OH)D levels. (e) BMI. (f) Habit of physical activities. VB, vitamin B1; BMI, body mass index; PA, physical activity.

**Fig. 3.**
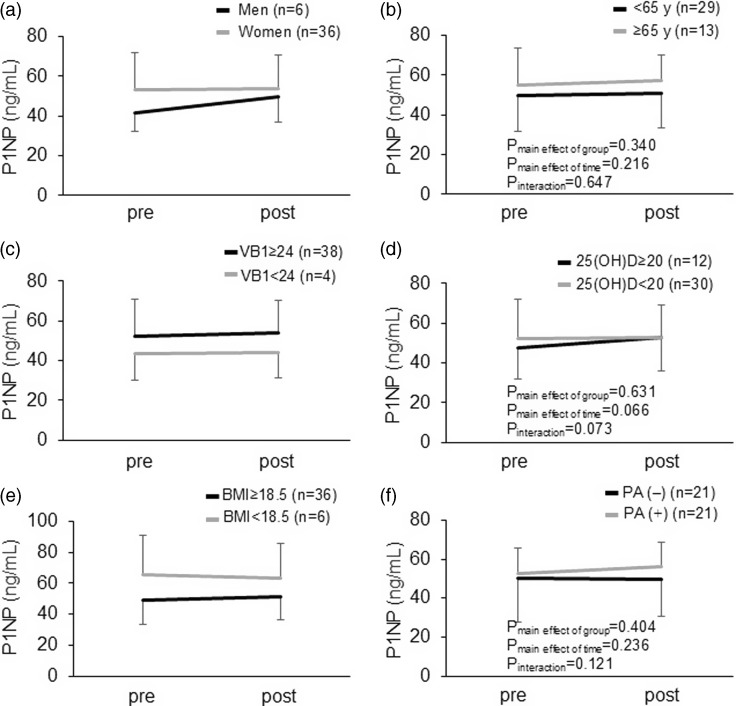
Subgroup analysis for the changes in the serum P1NP levels. (a) Sex. (b) Age. (c) Baseline VB1 levels in whole blood. (d) Baseline serum 25(OH)D levels. (e) BMI. (f) Habit of physical activities. VB, vitamin B1; BMI, body mass index; PA, physical activity.

The subgroup analysis in the total participants showed that no significant interaction was observed between the intervention period and baseline characteristics, including sex, age, BMI, and the baseline whole blood levels of vitamin B1 categories (Fig. 2a-f), in relation to the serum TRACP 5b concentrations, whereas significant interactions were observed between the intervention period and sex in relation to the P1NP levels (Fig. 3a).

## Discussion

In this study, we investigated the effect of vitamin B1 supplementation on the serum markers of bone turnover and found that TRACP 5b, a bone resorption marker, was reduced after 1 month of vitamin B1 supplementation at a dose of 28 mg/day in middle-aged and older Japanese adults. To our knowledge, this is the first clinical trial to implement the use of vitamin B1 supplements in humans.

This pilot trial enrolled community-dwelling Japanese adults without any severe comorbidities and used the usual dosage of vitamin B1 supplement recommended by the manufacturer. Because of the nature of the present study, we targeted healthy middle-aged and older adults among the general population to determine the potential efficacy of vitamin B1 in improving bone health through the measurement of the serum markers associated with bone turnover and metabolism. Based on this rationale, we selected participants without specific causes that have been recognised to affect bone turnover and metabolism, such as uncontrolled diabetes, chronic kidney disease, rheumatoid arthritis, taking medications including corticosteroids and vitamin supplements, as well as recent fractures.^(2,5,20)^ On the other hand, when vitamin D insufficiency and deficiency are defined as serum 25(OH)D levels of 12 to <20 ng/mL and <12 ng/mL, respectively,^(21)^ the frequencies of each condition were 38.1% and 33.3%, respectively. While vitamin D deficiency has been reported to be prevalent in most regions studied,^(22)^ in a previous survey of healthy adults living in two Japanese cities, the prevalence of vitamin D insufficiency and deficiency in a winter rural area were 38.9% and 33.3%, respectively,^(23)^ which is consistent with the results observed in the present study. Collectively, vitamin D insufficiency and deficiency, which may contribute to the development of osteoporosis and increased risk of fractures and falls in older adults, were common in the Japanese general population, especially among those who lived in rural areas.

In the present study, the serum TRACP 5b levels were reduced 1 month after vitamin B1 supplementation, suggesting the possibility that BMD and fracture risk may be changed, but further research would be needed to establish this. In a recent animal study, osteoporotic mice treated with a thiamine-rich diet showed increased bone strength compared with those treated with a thiamine-deficient diet, with in vitro evidence of regulating the receptor activator of nuclear factor κB ligand-mediated osteoclast differentiation.^(11)^ A clinical study also reported that thiamine deficiency was observed in admitted patients with femoral neck fractures.^(9)^ Recent studies have explored the clinical utility of TRACP 5b, demonstrating its effectiveness in monitoring treatment responses. For instance, it has been shown to correlate well with changes in bone mineral density (BMD), which is crucial for evaluating osteoporosis treatments.^(24)^ The impact of the mean change of 10.3% in the TRACP 5b levels from baseline as observed in the present study may not represent a minimum significant changes of 12.4%.^(25)^ Further research with longer observation periods and multiple doses is needed to determine the clinical relevance of vitamin B1 supplementation in bone health.

After 1 month of vitamin B1 28mg supplementation, the serum phosphorus and PTH concentrations did not change, whereas the corrected serum calcium concentrations increased and 25(OH)D concentrations decreased. In view of the physiological response of calcium metabolism, which is primarily regulated by PTH and the active form of vitamin D, the explanation for these changes in serum is unclear. A 5-year observational study of 75-year-old women reported no group differences in the PTH and 25(OH)D concentrations by the degree of bone turnover as defined by the serum TRACP 5b concentrations.^(26)^ Considering that this study was conducted mainly during the winter season in healthy middle-aged and elderly subjects whose dietary habits and other lifestyle habits were not expected to change, it is assumed that one of the reasons may be a decrease in serum 25(OH)D concentration due to the decreased production in the skin caused by lack of sunlight in winter.^(27)^ Previous studies conducted in two regions of Japan also support this theory.^(23)^ The long-term evaluation of bone metabolism markers, including 25(OH)D concentrations and serum-corrected calcium levels, which showed changes in this study, will be necessary when designing future clinical studies.

The subgroup analysis showed no interaction between vitamin B1 supplementation and the participants’ characteristics, including sex, age, and baseline whole blood vitamin B1 concentrations, in relation to the serum TRACP 5b levels, indicating that the association between vitamin B1 supplementation and changes in the serum TRACP 5b concentrations over 1 month was comparable among the participant characteristics at enrollment. Of these, we observed a trend toward an interaction between vitamin B1 supplementation and a change in serum TRACP 5b levels with sex (greater changes in men), as well as an interaction between vitamin B1 supplementation and a change in the serum P1NP levels. Based on the description suggesting that the effects of treatment on BMD and bone turnover may be similar in men with osteoporosis and postmenopausal women,^(2)^ sex differences in the effects of vitamin B1 in relation to bone turnover need to be further investigated.

However, this study has several limitations. First, this is a single-arm before–after study investigating the potential of vitamin B1 supplementation to modulate bone turnover in middle-aged and older adults. This simplified study design was deemed suitable for obtaining a preliminary proof-of-concept of efficacy, even under the limited conditions of study population and cost. Due to these restrictions, we were unable to assess the attributable effects of supplementation on the changes in bone turnover markers compared with controls who did not take vitamin B1 supplements. Second, the dosage of vitamin B1 was 28.0 mg/day only; the appropriate dose of vitamin B1 for the prevention or treatment of bone loss has not been determined. Third, for the convenience of the study participants, the timing of blood collection for bone turnover markers in this study was in the afternoon rather than the recommended fasting time.^(28,29)^ Under these conditions, the study was designed to minimise intraindividual variation in the bone turnover markers by timing the baseline and post-intervention blood collections within a fixed time window in the afternoon and by selecting serum TRACP 5b and P1NP, which are known to show relatively small diurnal variations among the bone turnover markers.^(29)^ In addition to the day-to-day variability of its concentrations, TRACP 5b has been reported to be unaffected by food intake.^(24)^ Despite these limitations, this pilot study provides suggestive evidence that vitamin B1 can be effectively administered to middle-aged and older adults without serious medical conditions to maintain and/or improve bone turnover.

In summary, we demonstrated that the serum TRACP 5b levels were reduced after vitamin B1 supplementation in middle-aged and older adults during the study period. The results of the present study may create a hypothesis that vitamin B1 is associated with bone health in humans. Further studies are needed to determine the effects and mechanisms underlying the impact of vitamin B1 on bone health and the differences in these effects by individual characteristics.

## Supporting information

Hara et al. supplementary material 1 Hara et al. supplementary material

Hara et al. supplementary material 2 Hara et al. supplementary material

## Supplementary material

The supplementary material for this article can be found at https://doi.org/10.1017/jns.2025.22
