# Supplementary material for: Effect of vitamin B1 supplementation on bone turnover markers in adults: an exploratory single-arm pilot study
Source: J Nutr Sci. 2025 May 8;14:e34. doi: 10.1017/jns.2025.22 (PMC12075007; doi:10.1017/jns.2025.22)
Supplement: Hara et al. supplementary material 1 — Hara et al. supplementary material [file S2048679025000229sup001.docx]

**Supplementary information**

‘Effect of vitamin B1 supplementation on bone turnover markers in adults: a single-arm pilot study’

Akinori Hara, Chie Takazawa, Hiromasa Tsujiguchi, Jiaye Zhao, Masaharu Nakamura, Tomoko Kasahara, Yukari Shimizu, Hiroyuki Nakamura

*Methods of measuring whole blood vitamin B1*

SRL Inc. have developed and utilized the quantitative analysis method of the whole blood vitamin B1 using LC-MS/MS (Miyagawa H, et al. *J Anal bio-science* 36, 327–330, 2013 [in Japanese]). This method shows good quantitative results in terms of reproducibility and validity. Furthermore, the analysis time is about just 4 minutes. Details regarding the measurement method are given below.

1. Procedure

1-1) Name of method

Liquid chromatography-tandem mass spectrometry (LC-MS/MS)

1-2) Measuring principle

The blood is deproteinized, hydrolyzed with acid phosphatase in order to convert phosphate ester form to thiamine, applied to a reversed-phase column, and then subjected to LC-MS/MS for determination of vitamin B1.

1-3) Reference value

i) Reference range

24～66 ng/mL

ii) Basis for setting

In-house setting at SRL, Inc.

2. Materials

2-1) Materials and volume required

Blood (with EDTA-2Na) 0.5 mL

2-2) Collection and storage conditions

i) Collection conditions

Use a special light-blocking tube containing EDTA-2Na

ii) Storage conditions

Under Freezing

3. Measuring equipment

| No. | Name of equipment | Manufacturer's name |
| --- | --- | --- |
| 1 | LC-MS/MS | Shimadzu Corporation |

4. Reagents

| No. | Name of reagents | Manufacturer's name |
| --- | --- | --- |
| 1 | mobile phase | In-house preparation at SRL, Inc. |
| 2 | acid phosphatase solution | In-house preparation at SRL, Inc. |
| 3 | Protein removal solution with internal standard | In-house preparation at SRL, Inc. |
| 4 | sodium acetate solution | In-house preparation at SRL, Inc. |
| 5 | Inertsil ODS-4 2.1×50mm | GL Sciences Inc. |

5. Measurement Procedure

Dispense specimen to plate

↓

Dispensing of protein removal solution with internal standard

↓

Centrifugation

↓

Dispense the supernatant into a plate containing sodium acetate

↓

Dispensing acid phosphatase solution

↓ Incubation under 37℃

↓

LC-MS/MS analysis

**Supplementary Figure 1. Participants’ enrollment and follow-up**

eGFR, estimated glomerular filtration rate
