# Supplementary figures and images for: Effect of vitamin B1 supplementation on bone turnover markers in adults: an exploratory single-arm pilot study
Source: J Nutr Sci. 2025 May 8;14:e34. doi: 10.1017/jns.2025.22 (PMC12075007; doi:10.1017/jns.2025.22)

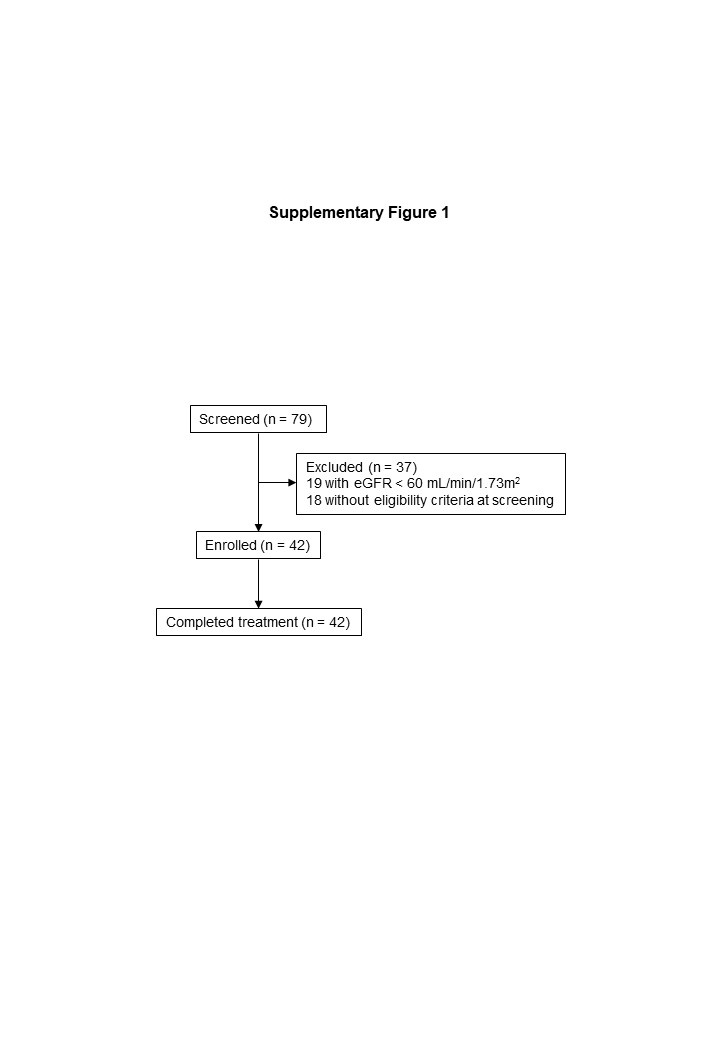

Supplement: Hara et al. supplementary material 2 — Hara et al. supplementary material [file S2048679025000229sup002.zip › Supplementary Figure1.jpg]
